# The cognitive cell: bacterial behavior reconsidered

**DOI:** 10.3389/fmicb.2015.00264

**Published:** 2015-04-14

**Authors:** Pamela Lyon

**Affiliations:** Southgate Institute for Health, Society and Equity, School of Medicine, Flinders UniversityAdelaide, SA, Australia

**Keywords:** cognition, evolution, signal transduction, memory, information-processing, valence, learning, communication

## Abstract

Research on how bacteria adapt to changing environments underlies the contemporary biological understanding of signal transduction (ST), and ST provides the foundation of the information-processing approach that is the hallmark of the ‘cognitive revolution,’ which began in the mid-20th century. Yet cognitive scientists largely remain oblivious to research into microbial behavior that might provide insights into problems in their own domains, while microbiologists seem equally unaware of the potential importance of their work to understanding cognitive capacities in multicellular organisms, including vertebrates. Evidence in bacteria for capacities encompassed by the concept of cognition is reviewed. Parallels exist not only at the heuristic level of functional analogue, but also at the level of molecular mechanism, evolution and ecology, which is where fruitful cross-fertilization among disciplines might be found.

## Background

### A New Look at an Old Idea

The idea that microbes might know their world, not merely bump into it, is controversial but unoriginal. At the beginning of the 20th century, Jennings (1905/1962) claimed the behavior of the ‘lower animals,’ notably paramecia but including bacteria, could tell us much about the behavior of the ‘higher animals,’ including humans. *The Animal Mind* (Washburn, 1936), the first US textbook on comparative psychology (first edition 1908), begins with amoeba. Biochemist Daniel Koshland provided the modern scientific equivalent of Jennings’ argument in an under-appreciated monograph on bacterial chemotaxis (CT) as a model system for the study of behavior, and remarked (following Pope) that “the proper study of mankind is the bacterium” (Koshland, 1980b). Philosopher of science Karl Popper went further and argued (only slightly tongue-in-cheek) that in the evolution of problem-solving “from the amoeba to Einstein is just one step” (Popper, 1999).

More recently, in view of tremendous advances in methods for studying individual cells as well as population-based microbial behavior, the bacterium has been compared explicitly to a parallel distributed processing (PDP) network (Bray, 2009) that displays ‘minimal cognition’ (Lengeler et al., 2000; van Duijn et al., 2006; Shapiro, 2007). Arguments concerning bacterial ‘intelligence’ (Jacob et al., 2004; Hellingwerf, 2005; Marijuán et al., 2010) and even cells ‘thinking’ (Ramanathan and Broach, 2007) are appearing in mainstream journals, including the special series in this one. British psychologist Richardson (2012), who has been researching human intelligence (sometimes despairingly) since the early 1970s, recently concluded in an extraordinary article in *EMBO* that the nascent study of unicellular intelligence might provide the key to understanding intelligence in complex vertebrates, including humans. Unknown to Richardson (2012), a microbiologist specializing in computational biology has introduced a plausible formula for establishing ‘bacterial IQ,’ based on genome size and proportion of DNA segments coding for signal transduction (ST) proteins, as well as a rough gage of ‘introversion’ or ‘extroversion’ based on the relative proportion of environment-contacting ST systems (Galperin, 2005). Finally, neuroscientists and neurobiologists tracing the evolution of complex human, brain-based behavior increasingly locate its origins in the microbial realm (Allman, 1999; Damasio, 1999; Greenspan, 2007).

Yet, while work on bacterial adaptation underlies the contemporary biological understanding of sensory ST (Wadhams and Armitage, 2004), and ST in turn provides the foundation of the information-processing approach to cognition that (rightly or wrongly) remains the hallmark of the ‘cognitive revolution’ (Miller, 2003), cognitive scientists are largely oblivious to research in this and other areas of microbial behavior that might provide insights into problems in their own domains. For example, some flagellated bacterial species, paradigmatically *Escherichia coli*, have been found to possess large clusters of interacting sensory receptors, typically at the leading pole of the cell, which function as complex information-processing units linked to motor activity (Hazelbauer et al., 2007) and have been dubbed by some a ‘nanobrain’ (Webre et al., 2003). Meanwhile, microbiologists seem equally unaware of the potential importance of their work to understanding cognitive capacities in metazoans including vertebrates.

In the hope of stimulating interest among microbiologists in this area, this review brings together contemporary evidence for cognition in eubacteria in core areas of cognitive research: sensory ST, valence, communication, sensorimotor coordination, memory, learning, anticipation, and decision making in complex and changing circumstances. The article will show that parallels exist not only at the heuristic level of functional analog, but also at the level of molecular mechanism, evolution and ecology, which is where fruitful cross-fertilization among disciplines might be found. Archaea, whose behavior is less well documented and appear (so far) to possess far fewer ST systems than bacteria (Ulrich and Zhulin, 2010), will not be discussed here.

The review is organized into two parts. Part 1 provides an introduction to the study of cognition. Part 2 sets out a proposed bacterial cognitive toolkit. Because there is a great deal of ground to cover, and not a lot of space to cover it in, the mechanisms of how systems work will be sketched in broad outline, not in detail, except in those cases where the details are either not widely known or not easily accessible in the literature. The aim here is to stimulate microbiologists to think about their work in the broader context of a cognitive biology (Kovác, 2000; Auletta, 2011; Bechtel, 2014; Fitch, 2014) and to consider designing experiments to explicitly enhance this understanding.

## Part 1. Cognition: a Biological Function Unlike Any Other?

Whether or not bacterial behavior ultimately should be described as ‘cognitive’ is a matter for the scientific community to decide, when consensus finally forms around what cognition is (Bechtel et al., 1998). The absence in psychology and elsewhere in the cognitive sciences of agreed definitions for key concepts, such as cognition, has been decried for well over a century – James (1984) arguably setting the benchmark for despair – and continues to be a cause for concern (Staats, 1983; Sternberg, 2005). As it stands, cognition, like ‘intelligence,’ is a highly polysemic theoretical construct that can be defined quite broadly (La Cerra and Bingham, 2002) or very narrowly (Wynne, 2004), depending on the investigator’s personal intuitions and the ground in which those intuitions are embedded, whether in the principles of biology or the human case (Lyon, 2006; see **Table 1**). Needless to say, this state of affairs is far from ideal.

**Table 1:** Broadly biological and narrowly human conceptions of the capacities encompassed by the general concept of cognition (Lyon, 2006).

Capacity	Biological (broad)	Human (narrow)
Affect	Valence: attraction, repulsion, neutrality (hedonic response).	Hedonic response plus conditioned or unconditioned cognitive appraisal (i.e., this is good/not good).
Sense perception	Ability to recognize existentially salient features of the external or internal milieu.	Conceptually mediated feature sensing.
Discrimination	Ability to determine that a state of affairs affords an existential opportunity or presents a challenge, requiring a change in internal state or behavior.	Ability to differentiate a state of affairs as a particular state of affairs and not another; having a concept.
Memory	Retention of information about a state of affairs for a non-zero period.	Retained information spatio-temporally decoupled from an immediate stimulus, which can be explicit/declarative or implicit/ procedural.
Learning	Experienced-modulated behavior change.	Classical conditioning; ability to change rules governing behavior.
Problem solving	Behavior selection in circumstances with multiple parameters and high degrees of uncertainty; adaptability.	Rational decision-making, abstract thinking.
Communication	Mechanism for initiating purposive interaction with conspecifics (or non-conspecific others).	Verbal or written symbol systems whose units have semantic content (meaning) and their deployment is organized according to rules (syntax), both of which are conventionally established expressly for the purpose of information exchange.
Motivation	Teleonomic striving; implicit goals arising from existential conditions.	Goal-driven behavior; goal is explicit (i.e., to the agent).
Anticipation	Behavioral change based on expectancy (i.e., if X is happening, then Y should happen).	Expectation based on past experience (potentially) explicit to the agent; planning.
Awareness	Orienting response; ability to selectively attend.	Reflexive awareness; ’what it is like to be X’.
Self-reference	Mechanism for distinguishing ‘self or’ like self from ‘non-self’ or ‘not like self’.	Self-awareness; concept of ‘self’.
Normativity	Error detection, behavior correction.	Value assignment based on experience, motivational state and/or convention.
Intentionality^∗^	Directedness toward an object.	Beliefs and desires.

^∗^A philosophically derived ‘mark of the mental’ popular in the 19th and 20th centuries (Crane, 1998).

The study of cognition as a biological function may be unique in the life sciences in the extent to which findings ultimately are calibrated against a single species, *Homo sapiens* (Shettleworth, 1993). The study of respiration and other biological functions, for example, are not so calibrated, although scientific investigation doubtless began with concern for the human case. (We stop breathing, we die – so what is breath?) Rather, evidence is followed wherever it leads, and it can lead to unexpected places. We know now that the oxidation of nicotinamid dinucleotide (NADH), the molecular substrate of cellular respiration, is a process universally shared, and in animals is relevantly similar across phyla. By contrast, prokaryotic memory was discovered more than four decades ago (Macnab and Koshland, 1972), yet is far from being accepted – even by microbiologists – as anything relevantly like memory in complex vertebrates, to say nothing of humans. Cognitive scientists might be similarly dismissive were they to give it any thought at all.

Memory, needless to say, is critical to cognition. Without memory, present circumstances have no context; the detection of change is impossible. Without the ability to detect change, the decision to alter behavior can only be random, haphazard. Without memory, learning of any kind is impossible. While cognitive scientists now accept that discoveries concerning the molecular basis of memory in the marine invertebrate *Aplysia* are relevant to the study of human memory (Kandel, 2006), they (to say nothing of microbiologists) have yet to connect the dots with memory processes in prokaryotes.

Nevertheless, similar ST mechanisms appear to be at work, albeit with different leading biochemical actors and different degrees of regulatory complexity. For example, ST in bacterial two-component systems (2CSs) relies principally on histidine kinases for protein phospho-transfer and DNA methylation, while genomic analysis shows that serine/threonine kinases, their analog in eukaryotes, comprise roughly 1 in 4 of the more than 500 protein kinases involved in ST in humans (Capra et al., 2006)^1^. However, analysis of bacterial genomes increasingly reveals serine/threonine kinases in prokaryotes, particularly those that live in changing environments and display complex social behavior, such as myxobacteria (Munoz-Dorado et al., 1993; Schneiker et al., 2007), *Bacillus subtilis* (Macek et al., 2007), and *Paenibacillus vortex* (Sirota-Madi et al., 2010). Calcium/calmodulin-dependent kinase II (CaMKII) is a serine/threonine kinase critical for memory formation in humans (Giese and Mizuno, 2013). Does it have a prokaryotic homologue? To my knowledge, no one is looking.

In short, in contrast to other life scientists, as well as to their forebears of yesteryear, contemporary cognitive scientists have been less inclined to follow the Darwinian principle – that biological functions differ substantially ‘in form but not in kind’ (Darwin, 1874/1909) – very far ‘down’ the phylogenetic yellow brick road in the search for clues as to the what and why of cognitive function (Bekoff et al., 2002).

Behavior generation lies at the heart of all cognitive inquiry, and while unicellular organisms are simpler than metazoans in structure, they are not always behaviorally less complex. The well-described social predation style, rippling behavior and fruiting body formation of *Myxococcus xanthus* arguably are more complex than the activities of most *Porifera* and even some worms with simple nervous systems. Extensive experimental evidence shows that microbial behavior is guided by processes that, in other contexts, are readily regarded as part of biological cognition, capacities which together encompass an organism’s ability to navigate, become familiar with, value, learn from and solve critical existential problems within its world of experience, including coordinating action with conspecifics. This may explain why, for the past several decades, microbial researchers increasingly have helped themselves to cognitive terminology (i.e., ‘decide,’ ‘talk,’ ‘listen,’ ‘cheat,’ ‘eavesdrop,’ ‘lure,’ ‘vote’) to describe complex bacterial behavior, often without caveat (e.g., Adler and Tso, 1974; Fuqua and Greenberg, 1998; Ben-Jacob et al., 2004; Bassler and Losick, 2006; Baker and Stock, 2007; Williams et al., 2007).

In the following pages I hope to show that such linguistic usage is not wholly metaphorical. I believe there is something going on at the microscopic level that doesn’t just ‘look’ cognitive, it *is* cognitive, or, more accurately, it is typically considered cognitive when studied in animals more like us.

### Biological Cognition

Discussions of cognition across phyla often degenerate into qualms about definition that typically begin with ‘yes, but what do you mean by…’ The concept that guides this investigation will be specified therefore. Because we are not concerned with the special capacities of a single mammalian species (i.e., *Homo sapiens*), a general definition from comparative psychology will serve, with some modification. Shettleworth (1998, p. 5) defines cognition as “the mechanisms by which animals acquire, process, store, and act on information from the environment. These include perception, learning, memory, and decision making.” Cognition thus is comprised of the total suite of mechanisms that underwrite information acquisition, storage, processing, and use. Although this definition can be applied to phyla Shettleworth (1998) may not have had in mind, e.g., microbes, it is (for the most part) uncontroversial within the domain of comparative psychology and cognitive ethology.

Shettleworth’s definition, and others like it in the cognitive scientific literature, emphasizes the psychologically delineated elements of the function. Others concerned with the specifically biological manifestation of cognitive function – which historically includes William James, H.S. Jennings, Jacob von Uexküll, Konrad Lorenz, James J. Gibson, and Humberto Maturana—emphasize the ecological context of information ‘use,’ what cognition does for the organism in the business of staying alive in a particular ecological niche. With this in mind, I extend the definition as follows:

Biological cognition is the complex of sensory and other information-processing mechanisms an organism has for becoming familiar with, valuing, and [interacting with] its environment in order to meet existential goals, the most basic of which are survival, [growth or thriving], and reproduction. (Lyon, 2014, p. 174)

While they have several forms of metabolism not found in any eukaryote (e.g., nitrogen fixation, methanogenesis), prokaryotes possess most of the biological functions shared by all animals, either by virtue of their own structure or those of their symbionts. Bacteria possess processes for breaking down and transforming nutrients into usable forms of energy for self-production or generating behavior, storing energy in molecules that can be catabolized when needed, and eliminating molecular waste generated during these processes. To stay alive, bacteria also appear to possess a ‘basic toolkit’ (Godfrey-Smith, 1996), which in other forms of life—especially mammalian life – would be termed cognitive. In the case of well-studied bacterial species (e.g., *E. coli*, *B. subtilis*, *M. xanthus*, *Pseudomonas aeruginosa*), the basic toolkit includes at least eight capacities found in different but recognizable forms in complex metazoans (see **Box 1**).

**BOX 1: The basic cognitive ‘toolkit.’**

**Table:** 

**Sensing/Perception**	The capacity to sense and recognize (re-cognize) existentially salient features of the surrounding milieu.
**Valence**	The capacity of an organism to assign a value to the summary of information about its surroundings at a given moment, relative to its own current state.
**Behavior**	The capacity of an organism to adapt via changing its spatial, structural or functional relation to its external or internal milieu.
**Memory**	The capacity to retain information about the immediate (and possibly distant) past, and to calibrate the sensorium to take account of this information, for example, via signal amplification.
**Learning**	The capacity to adapt behavior according to past experience, enabling a faster response time.
**Anticipation**	The capacity to predict what is likely to happen next based on an early stimulus.
**Signal integration (decision making)**	The capacity to combine information from multiple sources, because all organisms appear to sense more than one thing, and some bacterial species are equipped to sense dozens of different states of affairs.
**Communication**	The capacity to interact profitably with conspecifics, including initiating collective action, which may or may not include an explicit method of differentiating 'us' from 'them'.

Like all organisms, bacteria adapt to changes in their environment by modifying their metabolism and behavior. The traditional view is that this process depends on a limited number of highly canalized, inflexible mechanisms, which bear little resemblance to their behavioral counterparts in animals with nervous systems. However, evidence increasingly points to the idea that bacteria—particularly ecological ‘generalists’ as distinct from ‘specialists’—are highly social, flexible responders, responders (however limited their actual response styles) that rely on a sophisticated suite of sensory and information-processing mechanisms (Shapiro, 1998). Such mechanisms are characterized by non-linear responsivity, integration of multiple information channels, and (in the case of CT receptors, at least) habituation and adaptation phenomena. These structural and dynamical features are among the defining features of neuronal sensory processing in animals, including humans.

Keep in mind that a great deal of complexity will be obscured in the simplifications that follow. In each cell of *E. coli*, for example, which is not the smartest proteobacterium on the block (but no dummy, either), there are upward of 10,000 chemoreceptors per cell, each with multiple binding sites, whose output interacts with several flagella, on each of which are about 40 binding sites for CheY-P, the protein that modifies the direction of rotation. The behavioral output for a single cell is thus hugely complex, and much more refined (tumbles, twiddles, etc.) than normally described. When we simplify to get just two outputs, run and tumble, and then bundle behaviors to get population averages, a lot of the cognitive output is likely to be occluded. Consider an example with humans: if we assumed just two behaviors, walking and standing, and measured population averages using different stimuli, more would be hidden in the results than is revealed^2^.

## Part 2. The Bacterial Cognitive Toolkit

As do animals and plants, bacteria monitor the biotic and abiotic features of their surrounding milieu using a wide variety of sensory systems, and adapt their behavior and physiology in response to what they perceive in order to stay alive and reproduce. While the ecological niche of some bacteria is fairly stable, if sometimes extreme, many inhabit highly mutable environments and conduct highly complex lifestyles within them, which present considerable challenges, some of which are capable of being anticipated and others not. Like all organisms, bacteria do not gather information in a vacuum, but are able to assess the significance of the signals they receive relative to their own functioning, their internal milieu—although it is worth noting that the power spectrum of signals remains unknown for any bacterium^3^.

In the psychological literature, assignment of an existential value to the sum of information concerning current circumstances, internal and external, is called *valence*, which has been described as ‘a basic building block of emotional life’ (Barrett, 2006)*.* Valence refers to the attractiveness, acceptability or tolerability of a stimulus (Lyon, 2014). Usually such stimuli are rendered in terms of ‘positive’ or ‘negative,’ ‘attractant’ or ‘repellant,’ in addition to neutral stimuli to which the organism is indifferent, but these characterizations are not always stable. Aversive conditioning depends on the capacity of previously neutral or even positive stimuli to become negative for an organism under particular circumstances. Thus, all sensory signals have a context-dependent valence—positive, negative or neutral—which influences the available response.

In mammals specialized brain-based systems exist for this purpose, notably the hypothalamic-pituitary-adrenal (HPA) axis and the limbic system, which together appraise for a given signal not only *what it is* and *where it is* but also *what it means ‘for me’* (Vedder, 2008). This involves brain structures such as the amygdala and the orbitofrontal cortex. Although bacteria lack such specialized organs, the valence of a signal is implicit in the processes that coordinate, say, the rotation bias of flagellar motors or the complex developmental sequence of signaling, genetic transcription and protein expression that leads to sporulation.

One of the most resilient obstacles to thinking about microbes as potentially cognitive organisms is the lack of specialized sensory and information-processing systems that exist in vertebrates and especially mammals. In bacteria “sensory, regulatory, and metabolic networks must all drive environmental perception and corresponding action” within the boundaries of a single cell (Freddolino and Tavazoie, 2012; p. 364). In actual fact, while cognitive scientists for many years have tried to draw principled distinctions between ‘metabolic’ and ‘cognitive’ function (even in microbes—see, for example, van Duijn et al., 2006), this dividing line is increasingly difficult to defend even in human beings, given increasing understanding of immune system involvement in *normal* memory and learning (Yirmiya and Goshen, 2011) and the surprising effects of psychological stressors on metabolic physiology (Maier and Watkins, 1998; Yirmiya et al., 2006).

### Sensing: What’s Out there and What’s in Here

Bacteria are capable of sensing and responding to an astonishing variety of environmental signals: amino acids, sugars, oxygen, pH, osmolarity, temperature, light, secondary metabolites, molecular waste products (e.g., ammonia), environmental DNA, and other microbes (both conspecifics and other species)—even physical objects such as tiny, chemically inert beads (Dworkin, 1983).

Signal transduction systems in bacteria come in several basic forms that perform a wide range of behavioral and physiological roles (Ulrich and Zhulin, 2010): one-component systems (1CSs); 2CSs, which also come in hybrid forms; CT systems, a special sub-class of 2CSs; extracytoplasmic function (ECF) sigma factor proteins, the so-called ‘third pillar of bacterial ST’ (Staroń et al., 2009), as well as non-canonical systems that involve three and more components (Marijuán et al., 2010).

#### One-Component Systems

One-component systems consist of a single polypeptide chain containing both a sensor domain that registers a stimulus/signal and an output domain that elicits a response, for example, binding to DNA or interacting with other effector proteins. Thought to be evolutionarily older, 1CSs monitor the level of intracellular metabolites or respond to concentrations of membrane-permeable extracellular ligands (Galperin, 2005; Ulrich et al., 2005). Ulrich and Zhulin (2010) conclude on the basis of genomic data from thousands of species of bacteria and archaea that 1CSs are the most numerous by a considerable margin (see **Table 2**).

**Table 2:** Genome size and signal transduction, by millions of base pairs (Mbp) and type of system.

Organism	Genome (Mbp)	1CS	2CS	CT	ECF	OTHER	TOTAL
*Vibrio parahaemolyticus*	39.3	2,624	497	149	20	31	3,321
*Burkholderia xenovorans*	9.7	719	152	46	12	8	937
*Myxococcus xanthus*	9.1	315	263	60	41	8	687
*Escherichia coli* 0157	7.6	362	78	13	1	5	459
*Pseudomonas aeruginosa*	6.4	447	131	46	21	8	655
*Vibrio harveyii*	6.1	417	90	35	6	7	555
*Vibrio cholerae*	4.2	230	89	66	3	6	394
*Bacillus subtilis*	4.2	254	71	18	7	2	352
*Staphlococcus aureus*	3.1	119	34	–	–	2	155
*Mycoplasma genitalium*	0.6	5	–	–	1	–	6
*Buchnera aphidocola*	0.4	3	–	–	–	–	3

Two-component systems includes canonical types as well as those with hybrid components. Source: The MiST2 database: a comprehensive genomics resource on microbial signal transduction. Ulrich and Zhulin (2010) Nucleic Acids Research, doi:10.1093/nar/gkp940. (Accessed 21 Feb 2015 at http://mistdb.com/)

#### Two-Component Systems

Two-component systems segregate the sensor/input and effector/output functions between a sensory kinase (SK), which upon stimulation autophosphorylates, typically at a highly conserved histidine residue, and a response regulator (RR) to which the SK binds and transfers phosphoryl groups, sometimes at multiple sites. Phosphorylation and dephosphorylation, which occur at different rates at different sites, may affect transcription directly or as one step in a signaling sequence (Krell et al., 2010). The high-energy state created by modification of the RR constitutes a marker of environmental conditions at a particular time, which lasts until the chemical group is removed, thus constituting the basis of molecular memory (Koshland, 1977). Although 2CSs are commonly comprised of membrane-bound sensors interacting with the surrounding milieu and with one or more intracellular RRs, in some systems both components are located entirely within the cytosol (Krell et al., 2009).

#### Multi-Component Systems

Because 2CSs have two inherent advantages—multiple possibilities for control and graded signal amplification—they appear to form ‘phospho-relay systems’ with other regulatory components that employ transient phosphorylation as a means of transducing signals within the cell (Hellingwerf et al., 1995). Processes that support the creation of phospho-relay systems include cross-talk between (e.g., Mitchell et al., 2009) and/or constitutive convergence of pathways (e.g., Tagkopoulos et al., 2008). Thus a phospho-relay system may incorporate a single accessory protein, which recognizes the signal/stimulus and transmits the molecular information to the sensor kinase, making a three-component system (Krell et al., 2010). Other systems comprise several components. For example, *E. coli* has a seven-component signaling system for detecting orthophosphate (P_i_) and regulating the genes of the phosphate (*Pho*) regulon through the PhoR/PhoB 2CS, a paradigm in bacteria of a membrane-bound receptor controlling cytoplasmic gene expression (Hsieh and Wanner, 2010). P_i_ is the bacterium’s preferred source of phosphorus, which is essential for physiological functioning, not least due to its ubiquitous role in ST. The seven protein components include the PhoR histidine kinase and its cognate PhoB RR; four components of the ABC transporter Pst; and PhoU, a chaperone-like protein that inhibits PhoR/PhoB (Hsieh and Wanner, 2010).

#### Extracytoplasmic Factors (ECFs)

The third most abundant ST elements in bacteria belong to the family of sigma (σ) factors, which bind to RNA polymerase to promote genetic transcription, the basic ‘housekeeping’ form of which is present in all RNA polymerase holoenzyme complexes (Mascher, 2013). ECFs belong to the σ^70^ subfamily of alternative σ factors. The average bacterial genome contains six (Staroń et al., 2009), although some species contain none (e.g., *Streptococcus aureus*) while others contain many (e.g., 41 in *M. xanthus*; Ulrich and Zhulin, 2010). Alternative sigma factors possess different promoter-recognition properties, which increase the options for transcription under challenging conditions. As Gruber and Gross (2003, p. 441) put it: “The cell can choose from its repertoire of sigmas to alter its transcriptional program in response to stress.” This suggests significant heterogeneity may exist in sigma factor utilization between cells, based on individual history as well as growth stage, which may provide a source of selectable variability. Mascher (2013, p. 153) observes that the majority of ECF groups are phylum-specific, “reflecting the similar life style and physiology of a given group of related organisms.” The activity of many sigma is typically controlled, and normally suppressed, by a cognate anti-sigma.

The activity of alternative sigma factors can be quite complex. Induction of the general stress response in *B. subtilis* is mediated by σ^B^, which is activated by energy stress in “discrete stochastic pulses,” the frequency of which increases with increasing levels of stress (Locke et al., 2011). The σ^B^ circuit constitutes a highly sensitive, stochastically activated phosphorylation switch controlled by positive and negative feedback that can either amplify the circuit or switch it off. These properties—ultrasensitivity, stochastic switch-like behavior and the capacity for autoamplification—are characteristic of neural networks (Bray, 2009).

### Autoinduction: Indirect Sensing Via Proxies

Autoinduction is the process by which an organism synthesizes a class of molecules, called autoinducers (AIs), which stimulate a change in genetic expression in the organism itself when the molecules reach a threshold concentration (Miller and Bassler, 2001). The change may result in the production of other molecules that perform functions—classically, bioluminescence—or initiate a complex regulatory cascade that leads to a global transformation of the cell, as in sporulation. The term quorum sensing (QS) was introduced when it became apparent that genetic changes were induced at concentrations that seemed largely dependent on population density (Fuqua et al., 1994), although see the section on “Communication and Sociality” below. The supposition is that QS systems ensure that individuals do not act in ways that are too costly and unproductive when undertaken by one or a few cells. A few cells releasing a virulence factor will be swiftly attacked by a host immune system; bioluminescent molecules secreted by a lone symbiont will not be visible.

Autoinduction is widely considered to be a form of communication to facilitate cooperative behavior and is the basis for many collective activities, including: social motility, such as swarming (Daniels et al., 2004); production of secondary metabolites, such as virulence factors, bacteriocins, toxins, bioluminescence, degradative enzymes, exopolysaccharides, and pigments (Bassler and Losick, 2006); global changes of cell state, such as the transition from exponential growth to the stationary phase (Lazazzera, 2000), initiation of chromosomal replication (Withers and Nordstrom, 1998), and induction and regulation of sporulation (Shank and Kolter, 2011); lateral gene transfer (Lanka and Pansegrau, 1999); symbiotic mutualism (Visick and McFall-Ngai, 2000); pathogenic infection (Von Bodman et al., 2003; Asad and Opal, 2008); and biofilm formation, maturation and dispersal (Kjelleberg and Molin, 2002; Oppenheimer-Shaanan et al., 2013).

In different species AI systems may have widely differing regulatory components and molecular mechanisms. Commonly, however, AI concentrations are detected via ligand binding at membrane-bound receptors (2CSs) or via intracellular receptors that detect membrane-diffusible peptides (1CSs), and detection at threshold stimulates not only changes in the cell’s genetic transcription but also up-regulates AI production (Rutherford and Bassler, 2012). The signal is thus amplified.

Four QS systems have been identified as ‘canonical,’ two each for Gram-positive and Gram-negative bacteria (Rutherford and Bassler, 2012), although there are others. Several species operate two autoinduction systems, but some operate three, such as the bioluminescent *Vibrio harveyii* (Mehta et al., 2009) and the opportunistic pathogen *P. aeruginosa* (Jimenez et al., 2012). Waters and Bassler (2005) identify three types of “network architectures” for the operation of multiple QS systems: parallel, serial and antagonistic. Parallel systems may serve to filter out noise, either from non-signaling molecules in the environment or from signal mimics, operating much like multiple signatories on a bank account (Taga and Bassler, 2003). By contrast, the QS systems in *P. aeruginosa* are initiated sequentially, enabling the expression of different virulence factors at different times. In *B. subtilis* the QS network is based on two peptides that operate antagonistically, stimulating one of two mutually exclusive developmental programs, competence or sporulation, depending on context (Waters and Bassler, 2005).

The reason autoinduction is important from a cognitive standpoint is twofold. First, it increases the level of complexity of signal integration within the cell, and suggests there is a hierarchy of signaling values based on the concentrations of these molecules. Second, it involves the use of proxies for conditions that cannot be sensed directly, for example, population density or the physical properties of the surroundings. AIs thus may provide a paradigm case of biological ‘information’ that is conventional—deployed with a degree of arbitrariness, as in language—or, in the case of biological systems, evolved via natural selection to have the meaning it does (i.e., the genetic code; Smith, 2000).

In actual fact, all biological ST systems have this quality of assigned or evolved meaning, even those that directly sense features of the environment, once the signal is transduced into the cell. Protein interactions become what Millikan (2004) calls “pushmi-pullyu representations,” which simultaneously transmit *what is the case* and *what to do about it*. In the evolution of communication systems, this is the beginning of “primitive content” (Harms, 2004). This is not the only view of what constitutes communication and how it evolved, particular in microbiology (see Keller and Surette, 2006, for an alternative view), but it has a robust foundation in the study of language, comparative psychology, cognitive ethology, and philosophy of mind (Oller and Griebel, 2004).

### Communication and Sociality

The domain of communication—notably, the ascription of ‘cooperation’ or ‘sociality’ to collective bacterial activity—is where the interpretation of bacterial behavior begins to resemble debates in comparative psychology over whether certain animal capacities are ‘genuinely cognitive’ or not, where the benchmark is the human case (Shettleworth, 1993). Social behavior brings into play capacities traditionally conceived of not only as cognitive but also distinctly human: intelligence, the ability to communicate meaningfully with conspecifics in order to coordinate behavior, to distinguish between self and others, to interpret and track others’ behavior over time, and culture (De Waal and Tyack, 2003). Aristotle identified ‘calculative reason’ as the defining property of the human psyche precisely because humans live complex social lives (Aristotle, 2001). Sociality requires cooperation and coordination, not merely acting together. Not surprisingly, the evolution of sociality has been a hotly disputed area of theoretical discourse in biology for several decades (Segerstrale, 2001), and remains so today. (See, for example, Wilson and Wilson, 2007 and West et al., 2007 for two diametrically opposed reviews of the same subject.)

The contemporary case against the unwarranted ascription of sociality to microbes was provided by Redfield (2002) in a much-cited article raising important questions, which to that time had not been asked, about the nature and function of autoinduction. Research was proliferating into QS as a form of bacterial communication. The notion of bacteria as social, even multicellular organisms was gaining currency (Shapiro and Dworkin, 1997; Crespi, 2001), and increasing research into biofilms suggested that microbial social life might be quite cosmopolitan, involving mixed populations and featuring divisions of labor (Watnick and Kolter, 2000). Few questioned the social hypothesis of QS, or attempted to bring an evolutionary understanding to the discussion. (For an important exception, see Brown and Johnstone, 2001.)

Arguing almost entirely in evolutionary terms, Redfield (2002) warned against the uncritical interpretation of autoinduction as a form of bacterial communication facilitating social behavior. According to Redfield’s (2002, p. 365) somewhat narrow rendering of evolutionary theory, QS genes could only evolve in situations where individual cells that invest energetic resources for a shared benefit “reproduce better than cells using their resources selfishly.” Such individual fitness benefits, Redfield (2002) noted, had been neither queried nor tested. As an alternative Redfield (2002) advanced the ‘diffusion-sensing’ (DS) hypothesis, which proposed that individual cells synthesize AIs to ascertain the physical properties of their environment to calibrate regulatory and behavioral responses for their own benefit. Although anthropomorphism is never mentioned, the article’s last paragraph gestures obliquely in that direction:

We seem to be most prone to errors with those processes that most strongly distinguish us from bacteria—our sexuality and our sociality… perhaps because we are social animals, we find the idea that bacteria have evolved communication and cooperation very appealing (p. 369).

An influential review of bacterial communication 4 years later endorsed the evolutionary approach, and drew a terminological distinction between ‘signals,’ molecules ‘evolved for’ the purpose of transmitting information between a sender and a recipient (which according to the authors constitute communication), and mere ‘cues,’ molecules that acquire a role in a bacterial behavioral economy but are not positively selected to be signals (Keller and Surette, 2006). These articles helped to inspire theoretical and empirical work seeking alternative explanations for autoinduction beyond the default assumption that QS was communication.

It did not take long for proliferating proposals to generate a terminological quagmire. Thus the ‘semantics of QS’ (Platt and Fuqua, 2010) and ‘the confusion about diffusion’ (West et al., 2012) became subjects for detailed investigation. **Table 3** summarizes alternative terms for autoinduction suggested by different researchers based on the dominant factor believed to be influencing concentrations of molecules in particular cases (Platt and Fuqua, 2010). Although they approach the issue in very different ways, Platt and Fuqua (2010) and West et al. (2012) draw similar conclusions that are relevant here.

**Table 3:** Alternative terms for autoinduction based on dominant factors influencing concentrations of AI molecules.

Term	Factor
**Spatial positioning**
Quorum sensing	Local population density
Positional sensing	Spatial organization of cells
Cluster sensing	Spatial organization and distribution of cells in clumps
**Diffusion properties**
Diffusion sensing	Diffusion rates
Compartment sensing	Limited diffusion due to enclosure
Confinement induced QS	Limited diffusion due to enclosure
**Spatial and diffusion properties**
Efficiency sensing	Local cell density and diffusion rates
Cumulative gradient sensing	Accumulated cue as determined by cell density, production rate, and diffusion rate
**Environmental conditions**
Diel sensing	Cue stability due to pH conditions that vary periodically

(Source: Platt and Fuqua, 2010, pp. 18, 383–387 trends in microbiology).

First, both agree that some forms of autoinduction enable social behavior in bacteria, and can be appropriately viewed as communicative. Both cite experiments in *P. aeroginosa* as the only explicit attempts to test the social-signaling hypothesis, and these findings support the hypothesis. Second, both agree that terminological expansion has gone too far, and that QS is now so entrenched that it can be used generically, with the understanding that usage carries no social implications in the absence of further investigation.

However, from a theoretical standpoint, considerable work remains to be done, mainly because evolutionary theory is far from univocal on the issue of how social behavior and communication evolve. A treatment of this topic is beyond the scope of this article. I have argued elsewhere that bacterial sociality has much to teach evolutionary biologists as they refine the overarching framework of evolutionary theory, which is still very much a work in progress (Lyon, 2007).

### Lifestyle Complexity = Signaling Complexity

There is a strong positive correlation between bacterial genome size and the number of different ST proteins a microbe can synthesize (Ulrich and Zhulin, 2010), as well as between the complexity of a bacterium’s lifestyle and the number of ST pathways available to support its behavior and physiology (Galperin, 2005; Ulrich et al., 2005). Similarly, ECFs tend to be ‘under-represented and often absent’ in smaller bacterial genomes and ‘over-represented’ in bacteria with more complex lifestyles (Mascher, 2013). Moreover, there appears to be a correlation between the complexity of the signaling pathways a bacterium possesses and the complexity of its behavior, although more research is needed to see if this observation holds generally.

According to the microbial signal transduction (MiST2) database (Ulrich and Zhulin, 2010), from which all of the following figures are derived, the largest and smallest genomes in the bacterial kingdom are found among the gamma-proteobacteria (see **Table 2**.) The genome of *Buchnera aphidocola*, a symbiont of aphids, is a mere 400,000 base pairs (0.4 Mbp), which express only three (3) 1CSs and no ECFs. The largest genome, weighing in at an imposing 39.10 Mbp, belongs to *Vibrio parahaemolyticus*, a pathogenic species that inhabits brackish saltwater and has the capacity to withstand digestion both by seafood and humans. *V. parahaemolyticus,* about which comparatively little is known, expresses 3,321 ST systems, 79% of which (*N* = 2,624) are 1CSs.

In contrast, consider the delta-proteobacterium *M. xanthus*, a social predator that inhabits soil, arguably one of the most complex ecosystems on the planet. Potentially the primate of the eubacteria, *M. xanthus* is renowned for its myriad collective behaviors, including structured, multidimensional swarming motility (Kaiser and Warrick, 2014), pack-like predation (Berleman and Kirby, 2009), and the use of chemical cues to lure faster-moving prey (Shi and Zusman, 1993), as well as a complex developmental sequence leading to fruiting body formation and sporulation. At 9.14 Mbp, the *M. xanthus* genome is one of the top 20 in size and expresses 687 ST systems, of which more than half (54%, *N* = 372) are 2CSs. To date no other species, even those with complex ways of life, appears to have such a large proportion of 2CSs systems (see **Table 2**).

In addition, *M. xanthus* has twice the number of ECFs as *V. parahaemolyticus,* providing greater flexibility in responding to stress (Ulrich and Zhulin, 2010). Moreover, it has an unusually large number of genes (97) involved in synthesizing serine/threonine protein kinases (Goldman et al., 2006), signaling proteins that are rare in prokaryotes but which are involved in a wide variety of processes in unicellular and multicellular eukaryotes. Inhibitors for eukaryotic protein serine, threonine and tyrosine kinases have been shown to inhibit development in *M. xanthus* to varying degrees.

Two decades ago, philosopher of science Godfrey-Smith (1996) proposed the environmental complexity thesis to explain ‘the function of mind nature.’ Godfrey-Smith (1996) advanced the idea that the evolutionary ratchet driving cognition to more complex and powerful forms in some biological lineages is the complexity of the ecological niche they inhabit. While Godfrey-Smith did not have microbes in mind (see, in particular, Godfrey-Smith, 2002), it may be that bacteria provide the best test case of his thesis’s fundamental assertion.

### Motility

Chemotaxis is the directed movement of one or more cells toward external conditions the organism(s) determine are more favorable for growth and survival, and away from conditions determined to threaten survival (Webre et al., 2003). Although often studied and described in terms of discrete ‘attractants’ and ‘repellents,’ the assessment of valence by the cell that underlies CT in the presence of both has long been known to be non-linear even in simple laboratory setups (Adler and Tso, 1974), to say nothing of ecologically valid conditions. Bacterial motility takes several forms, but flagellar CT in *E. coli* was the first bacterial system of motility to be characterized in detail. Genes for the highly conserved methyl-accepting proteins of the *Che* system are genomic signposts for identifying CT systems in other bacteria (Ulrich and Zhulin, 2010).

Nevertheless, a stream of recent research into swarming motility in *M. xanthus* (Mignot et al., 2005, 2007; Nudleman et al., 2005; Mauriello et al., 2009b; Nan et al., 2010; Kaiser and Warrick, 2011, 2014; Pathak et al., 2012) has thrown light on very different processes of motility that involve vastly more components and produce much more observable behavior than flagellar rotation mediated by *Che* proteins.

Myxococcal swarming displays compelling similarities to flocking in birds, shoaling in fish and swarming in insects, the mechanisms of which have yet to be resolved in these animals (Wu et al., 2009). Other features appear to have mechanistic and functional similarities to critical signaling pathways involved in development in animals, notably, Wnt and Hedgehog (Kaiser and Warrick, 2014). Yet another feature—‘resetting’ a cytoplasmic pacemaker that regulates periodic reversal in cell direction (Kaiser and Warrick, 2014)—appears (to this writer) to bear a functional resemblance to synaptic scaling in neurons, a process critical to memory and learning in animals with nervous systems (Turrigiano, 2008).

Swarming motility in *M. xanthus* has long presented challenges to researchers. Swarming uses two types of motility system, initially believed to be for different styles of life, (S)ocial or (A)dventurous (Spormann, 1999). S-motility, the mechanism of which has been known for decades, involves type IV pili at the leading pole of the cell that retract, pulling the cell forward. A-motility, on the other hand, has defied adequate characterization, despite identification of about 40 genes involved in its implementation (Nan et al., 2010). However, recent research by Kaiser and Warrick (2014) using fluorescent protein labeling and time-lapse microscopy has illuminated the role of A-motility in swarming, and helped to make sense of important work over the previous decade by several research groups.

This is what was known about swarming motility in *M. xanthus* before the time-lapse work. First, it is collective, involving large numbers of cells, and relatively structured. Propelled by type IV pili, individual cells tend to align with one another. A large-scale manifestation of this is rippling, believed to be a strategy for distributing nutrients from lysed prey (Berleman et al., 2008). Although the proteins involved in myxococcal swarming are homologues of the classic CT proteins, the motility is not chemotactic (Dworkin and Eide, 1983; Kaiser and Warrick, 2011), which shows how similar proteins can function quite differently in similar contexts.

Second, cells in a swarm are constantly moving, periodically reversing direction in a cycle optimized to roughly 8 min, which appears to decrease the probability of collision as in shoals of fish or flocks of birds (Wu et al., 2009). According to a computational model developed to generate predictions about periodic reversal, an 8-min reversal interval results in cell orientations correlating over 10 cell lengths (≈50 microns), whereas a 50-min reversal interval results in correlation over only three cell lengths, and only one cell length with no reversal. Experimental observation aligns so closely with the model’s predictions that the probability of positive natural selection is high (Wu et al., 2009).

Cell reversal appears to be somehow obligate to myxococcal life, not simply to motility. In the hundreds of motility mutants created since 1970, none to date has been unidirectional (Kaiser and Warrick, 2011). This may be because the continuous movement of cells in a swarm is necessary to facilitate access to oxygen (above) and nutrients (below), and thus to reduce intercellular competition (Kaiser and Warrick, 2011).

Third, the gliding motility of non-motile cells can be restored through complementation (Velicer et al., 2002). Whereas in other social species, (e.g., honeybees, primates) ‘cheaters’ or ‘freeloaders’ are punished to keep them cooperating for collective benefit (Putterman, 2010), in swarming *M. xanthus* they appear to receive assistance. Complementation takes place through the efficient physical exchange between two cells of outer membrane lipoproteins necessary for motility, the S-motility protein Tgl and the A-motility protein CglB (Nudleman et al., 2005; Pathak et al., 2012).

Exchange is effected through the alignment and transient attachment of focal adhesion clusters (FACs), complexes of at least 15 known proteins located on the sides of the cell’s leading half—not the leading pole, as in CT (Mignot et al., 2007). When cells reverse direction the FACs retain their position on the leading half by relocating to the trailing half, along with S-motility proteins and type IV pili (Mignot et al., 2005, 2007). In short, the motility apparatus of the cell is constantly changing position during swarming.

Cell reversal, together with S-motility and FAC relocation during cell reversal, are regulated by a ‘pacemaker’ protein cluster located in the cytoplasm comprising three proteins of the Frz chemosensing pathway, the receptor for which is FrzCD (Kaiser and Warrick, 2011). The size, number and location of the Frz-clusters are constantly changing, or oscillating, and when cells make side-to-side contact, the Frz-clusters transiently align (Mauriello et al., 2009a). Frz-cluster number correlates with the frequency of cellular reversal: fewer clusters are found in hypo-reversing mutants, while more clusters are found in hyper-reversing mutants (Mauriello et al., 2009a). Wu et al. (2009) hypothesize that the *frz* genes “are part of a reversal clock” that operates a “reversal switch” driven by the small G protein MglAB, which induces the change in gliding direction. How the cytoplasmic pacemaker is connected to the cell surface remained unclear, although Nan et al. (2010) described a complex of proteins required for gliding that spanned the cytoplasm, inner membrane and periplasm and included the A-motility protein CglB.

Finally, swarms are different from colonies. Swarming motility depends on growth—it ceases when growth ceases—but swarms expand mainly by movement, not growth (Kaiser and Warrick, 2011). Although cells in a swarm are in all stages of the growth cycle, only 10% of swarm expansion is shown to be due to growth, whereas in a colony 90% of expansion is growth-related (Kaiser and Warrick, 2011).

The remainder of the section, which is derived entirely from Kaiser and Warrick (2014), describes what time-lapse microscopy of cells with fluorescent-labeled proteins revealed about what goes on in myxococcal swarms.

First and foremost, swarming *M. xanthus* don’t just move, they build two types of structures, the function of which are unclear: planar rafts, which have flat tops, and rounded mounds, which have five layers. Periodically, the cells on the top layer of the mounds translocate in an explosive, synchronized descent to the peripheries of the fourth layer. The explosive descent appears to be the result of a synchronized and simultaneous inhibition of all motility engines, A and S. From their new position the translocated cells slowly and asynchronously begin the climb to the top again, using pili-driven S-motility. When they have reached the top layer, the cells align into a compact arrangement as a result of A-motility signal proteins. The entire process takes about 14 min.

The mechanism by which this occurs is unknown, but what appears to take place is this. At low cell density, the FACs assemble on the sides of the leading half of the cells. The FACs then vanish and relocate to the trailing half, which upon cell reversal becomes the leading half. At high cell density, cells transiently attach to one another, side-by-side, as they move in the opposite direction along their long axes, bringing the FACs into alignment.

Kaiser and Warrick (2014) hypothesize that pairing facilitates passage of a signal between cells involving CglB and other A-motility proteins (as per Nudleman et al., 2005; Pathak et al., 2012). Supporting earlier observations of a motility complex, CglB proteins are found in all of the compartment spaces of the bacterium from the outer member to the periplasm and peptoglycan succulus to the cytoplasm, where the Frz-cluster pacemakers are located (Kaiser and Warrick, 2014). If the mechanism operates similarly to Wnt signaling in eukaryotes, as the researchers propose, the four levels of layering could allow Cglb proteins to pass the signal sequentially from one compartment space to another, providing a high degree of specificity between the upstream and downstream binding partners as well as with the intracellular compartment.

The signaling synchronizes the Frz-cluster pacemakers in the cell pair, which brings them into phase, effectively ‘resetting’ both cells to the average value of their pre-connection phases, a mechanism reminiscent of the scaling of synaptic firing potentials in neurons to ensure that overstimulation of one synapse does not distort the functioning of the network (Turrigiano, 2008).

Pairing is followed by cell reversal, which allows the cells to separate and move in a new direction, thus enabling the signal (whatever it is) to be spread, and the cells to become synchronized, which is necessary for coordinating the explosive descent. Thus, as Kaiser and Warrick (2011, p. 1309) observe: “Every cell in a mound should be available for pairwise signaling, cells in each layer are in contact with one another, and cells in a mound are able to move from one layer to another.” CglB is critical to this dynamic process of construction: CglB mutants can swarm but they cannot build.

In short, the sensorimotor activity of *M. xanthus* is astonishingly complex, and understanding this behavior may throw light on the sensorimotor behavior of social animals, such as birds, fish, and insects.

### Predicting What Comes Next: Memory, Learning and Anticipation

Traditionally it was thought that, however, complex their signaling machinery, all bacteria can do is sense and respond to ensure that existential parameters stay within the range of tolerance for survival and reproduction (Freddolino and Tavazoie, 2012). A growing body of evidence in the past decade suggests that bacteria are not simply backward-looking responders to stimuli already past—an assumption long-discredited in animals (Cahill et al., 2001)—but are dynamic predictors actively oriented toward what comes next (Tagkopoulos et al., 2008; Mitchell et al., 2009; Goo et al., 2012).

In all animals and plants (Trewavas, 2014), the ability to predict is predicated on memory and learning, which can take place within the life of an organism or across generations through epigenetic inheritance (Jablonka and Raz, 2009), very likely the basis of many evolved traits. Circadian entrainment of physiological processes to the planet’s most regular large-scale change—the daily cycle of light and dark—is a well-known class of evolved eukaryotic mechanisms that displays this anticipatory property. Thus far the only prokaryotes found to possess a proper circadian clock are photosynthetic cyanobacteria (Ouyang et al., 1998; Dvornyk et al., 2003), in which the phosphorylation state of *kai* proteins, not genetic transcription and translation, constitute the memory of elapsed time (Nakajima et al., 2005). The hemi-methylated state of DNA just after replication is an example of a genetic substrate for memory^4^. The key question, then, is whether and how bacteria remember and learn, and to what extent these capacities are relevantly like memory and learning in more complex animals.

#### Memory

Memory, typically the capacity to retain information acquired during the organism’s lifetime for a period sufficient to affect behavior, is part of the bedrock of cognition (Hilgard, 1980). For centuries memory, especially in its long-term modes, has been intrinsic to the anglophone concept of ‘mind’ (Earle, 1881). As stated earlier, the idea that bacteria exhibit some form of memory, or ‘memory effects,’ is now four decades old (Macnab and Koshland, 1972; Adler and Tso, 1974; Brown and Berg, 1974).

In his seminal explication of the RR model, Koshland (1977) describes the duration of protein phosphorylation as a bacterium’s memory because the period of modification effectively holds information about the state of the environment at a particular time, and the capacity to retain information, however, fleeting, is “no less real or useful to the bacterium than the memory of humans is to their behavior.” On the model proposed by Koshland (1980a), bacterial memory is “encoded in the enzymes that form and remove the RR.” Importantly from the standpoint of comparison with animals, the temporal duration of bacterial memory is not fixed, but is constituted (in CT proteins at least) by the difference between “a fast excitation process and a slow adaptation process,” which depends crucially on the strength and salience of the stimulus and the pathway involved (Morimoto and Koshland, 1991). The duration of response memory thus varies greatly among different chemotactic proteins, from seconds to more than 5 min (Yamamoto et al., 2005), the observational limit in the cited study. Moreover, chemotactic proteins may have multiple modifiable sites, suggesting the possibility of signalling and memory at different time-scales even within a single pathway (DeFranco and Koshland, 1980).

Morimoto and Koshland (1991) proposed a physiological distinction between short-term and long-term cellular memory, using the RR concept as a memory parameter. In the model *short-term memory* occurs when “stimuli evoke a transient elevation of the [amount of the] RR” in the cell, which “gradually returns to its initial value” when the stimulus is removed. *Long-term memory* occurs when “presentation of a second stimulus occurs before the initial response has returned to the baseline,” and “[s]uch stimulation results in a successive increase in the RR, which takes longer to return to baseline” (p. 2061). In Kandel’s work with *Aplysia*, the neuronal comparison that inspired Koshland, small-molecule neurotransmitters act rapidly because they are degraded and removed from the synaptic cleft by neuronal reuptake mechanisms, whereas neuropeptides passively diffuse away, and have longer-lasting effects (Kandel and Abel, 1995).

While protein dynamics are clearly important in memory processes in vertebrates and invertebrates, long-term memory in mammals, including humans, requires stable changes in neuronal structure. DNA methylation is now recognized as the mechanism by which such changes occur (Day and Sweatt, 2010). DNA methylation in general and chromatin remodeling in particular are also the mechanisms by which epigenetic learning takes place in vertebrates and invertebrates, allowing an organism’s experience in one generation to be incorporated into the adaptive repertoire of subsequent generations (Jablonka and Raz, 2009), particularly in relation to stress (Franklin et al., 2010). Epigenetic inheritance has been shown to modify stress responsiveness of bacteria (Veening et al., 2008; Chen et al., 2014).

Significantly, because stressful circumstances require immediate remedial action in a way attractive circumstances do not, in humans and other mammals memories for adverse events tend to be much stronger than those for positive events (Baumeister et al., 2001), which is why aversive conditioning is such a powerful tool.

#### Learning

In humans short-term and ‘working’ memory ranges from seconds to minutes, long-term memory from hours to decades (Terry, 2005). Long-term memory is often indistinguishable from non-associative learning, a type of learning in which “presentation of a particular stimulus alters the strength or probability of a response according to the strength and temporal spacing of the stimulus” (Rosenzweig et al., 1996, p. G-17). Non-associative learning includes *sensitization*, the amplification of a response following presentation of a stimulus, and *habituation*, the attenuation or extinction of a response to a stimulus upon repeated presentations (Shettleworth, 1998). Habituation and sensitization have both been demonstrated in bacterial CT (Koshland et al., 1982; Stock, 1999; Porter et al., 2011)—a discovery that “gave some neurophysiologists apoplexy, because they believed that a nervous system” was required (Taylor, 2004, p. 3761).

The operation of artificial and real neural networks depends on memory and non-associative learning effects, and the idea began to circulate that the ST systems of a bacterial cell might function as a neural network (Bray, 1990, 1995, 2009; Hellingwerf et al., 1995). Signal amplification is required for learning by neural networks, so a group of Dutch researchers decided to test the idea that *autoamplification* of genes in certain 2CSs, which further stimulates ST component production, might result in learning effects (Hoffer et al., 2001). Memory storage in animals with nervous systems was long thought to involve a mechanism involving an autophosphorylating protein kinase and paired phosphatase that operate together as a bistable switch (Lisman, 1985).

The *pho* regulon of *E. coli*, which operates to detect the metabolically critical nutrient P_i_ through the canonical PhoR/PhoB 2CS, was selected as the signaling pathway for experimentation (Hoffer et al., 2001). Upon stimulation the histidine kinase PhoR autophosphorylates and transfers phosphoryl groups to the cognate RR PhoR, which targets the *pho* operon, which then amplifies production of the two components. Thus signaling through PhoR/PhoB boosts production of the transduction components.

Cells from an exponentially growing population of *pho*A mutants were incubated first in a P_i_ limited medium (for 45 min at 42°), then in a high P_i_ medium (for 1 h at 30°). Later, the cells were transferred to P_i_ limited medium and incubated at 30°. The speed at which alkaline phosphatase production was induced, enabling the cell to scavenge traces of P_i_ or phosphorylated compounds from the environment, was then measured.

As hypothesized, the cells previously incubated in the P_i_ limited medium responded to the new limiting conditions faster than the control cells incubated exclusively in the high P_i_ medium. The faster response time correlated with the accumulation of ST components, a good probability that the response was the result of operon autoamplification rather than “a consequence of unspecific physiological effects.” Moreover, the learning behavior was “mechanistically and effectively different from the adaptation effects observed in CT,” and appeared to resemble immune system learning (Hoffer et al., 2001).

Another series of experiments involving *B. subtilis* are similarly indicative of non-associative learning. Wolf et al. (2008) selected *B. subtilis* for its sensitivity to environmental conditions and the well-known mechanisms governing sporulation. These mechanisms, triggered as part of a stress response, exhibit switch-like bistability, the basis of memory effects in computers, some physical compounds (i.e., magnetized iron), and neuronal activity in animals. Bistability is believed to have a role in human memory, particularly working memory (Durstewitz et al., 2000), but it has also been implicated in memory-associated processes in cellular plasticity and differentiation, e.g., in bone marrow (Wang et al., 2009).

Sporulation is an irreversible, energetically costly process of morphological and functional differentiation initiated as a last resort under starvation conditions (Dworkin and Losick, 2005) or a ‘bet-hedging’ strategy when nutrient supplies deteriorate (de Jong et al., 2011). The developmental sequence unfolds over 8 h, and results in the formation of specialized cells resistant to heat, chemical and UV stress. This involves the death of a significant proportion of the population, so the existential ramifications of movement toward sporulation are profound (for the survival of individual cells, at least), even before commitment becomes irreversible (Gonzalez-Pastor et al., 2003).

Three different strains of *B. subtilis* were grown under 10 different conditions, which varied according to the nutrient richness of the growth medium (rich and less-rich) and the density to which the population was allowed to grow, of which there were five classes (Wolf et al., 2008). In addition to a wild-type control, two fluorescent ‘reporter’ strains were created to measure three variables: (1) commitment to sporulation, a bistable process indicated by SpoIIE expression; (2) synthesis of AprE, a degradative enzyme that breaks down food sources, functions as a nutrient locator in deprived conditions, and shares many regulators with sporulation, but has a different expression pattern and is not believed to be bistable; and (3) growth, an indicator of general fitness.

After the growth period, the 30 different cell populations were sorted into identical densities and exposed to a common stressor, a starvation salt medium known to induce sporulation. Every 15 min for 24 h the 30 samples were measured in relation to growth, AprE synthesis, and commitment to sporulation (SpoIIE expression). Although Wolf et al. (2008) defined short-term memory effects as those measured within the first 11 h following exposure to the stress medium, in humans this timescale already would be said to involve long-term memory.

The study found that differences in life history gave rise to differences of varying strength in patterns of behavior relative to three variables (SpoIIE expression, AprE synthesis and growth) following a common stressor. Some of these effects lasted up to 36 h, far beyond the measurement period, which could either indicate ‘lifelong’ memory or the beginning of epigenetic memory across generations.

The data also suggest that memory strength and persistence are highly correlated with the energetics of survival. Growth medium was a better predictor of subsequent behavior than the density to which the original population was grown. Cells grown on the less-rich medium prior to exposure to the starvation salts pursued a more aggressive strategy. On average they grew “very fast” after exposure, induced AprE synthesis “after a brief lag,” and continued to grow even as the sporulation machinery switched on. By contrast, cells grown in the richer, Luria broth medium “seem[ed] to adopt a wait-and-see strategy,” did not grow and delayed sporulation and AprE synthesis “for many hours.”

As noted earlier, the finding that sporulation dynamics triggered by worsening circumstances exhibited “the most memory” (Wolf et al., 2008), despite involving the most energetically costly and far-reaching changes in the population, is consistent with results in human psychology and animal behavior (Lyon, 2014). Similarly, recent studies demonstrating anticipatory behavior in bacteria involve responses to predictable stressors.

#### Prediction

The ability to anticipate and pre-emptively respond to regular changes in the environment confers a considerable fitness advantage, and has been observed in bacteria quite apart from circadian periodicity. Fruiting body formation in *M. xanthus* in the presence of nutrients from prey displays a predictive quality (Berleman and Kirby, 2007). Fruiting body formation was long assumed to be a response to the stimulus of starvation, because that is how it is commonly induced for experiment, but Berleman and Kirby (2007) found that a mere ‘step down’ in nutrients, not depletion, is sufficient to initiate the process, which ceases if nutrient availability increases. Nevertheless, only recently has anticipatory behavior been explicitly tested in bacteria using as cues predictable environmental changes across the life cycle.

*Escherichia coli* inhabits several ecological niches during its life cycle, from water, sediment and soil to the mammalian gastrointestinal tract (Mitchell et al., 2009). To determine whether *E. coli* signaling networks are capable of predictive behavior “in a fashion similar to metazoan nervous systems,” Tagkopoulos et al. (2008, p. 1313) tested strains under conditions mimicking the transition from the outside world to the gastrointestinal tract of a mammalian host. As they enter the oral cavity cells immediately experience rising temperatures up to 37°. As they transition to the gut available oxygen drops precipitously to anaerobic conditions. If the homeostatic (sense-respond) framework is correct, *E. coli* should not repress respiration until a drop in oxygen is detected. On the other hand, if enteric bacteria are capable of dynamic predictive behavior, rising temperatures should induce respiratory repression.

This is precisely what the studies showed. Exposure to temperature upshift, from 25° to 37°, not only induced the heat shock response regulon, but also strongly repressed genes encoding components of the molecular machinery for aerobic respiration, rapidly reprogramming to anaerobic mode. Similarly, a downshift in temperature (mimicking the organism’s excretion from the host) initiated the return to aerobic respiration. The two responses are so tightly coupled that strains were evolved in the laboratory to reverse the sequence of expression; a downshift in oxygen activated the heat shock response in the evolved strain (Tagkopoulos et al., 2008).

Tight coupling of the two pathways in combination with susceptibility to alteration in the sequence of expression suggests a high probably of crosstalk between the signaling pathways. In the second series of experiments, crosstalk between two metabolic pathways in *E. coli* were not merely decoupled but completed dissociated (Mitchell et al., 2009). These discoveries would have been impossible without taking the organism’s habitat and ecology into account (Tagkopoulos et al., 2008).

In the mammalian GI tract *E. coli* is exposed to nutrient sugars in a sequence: first lactose, then maltose. Mitchell et al. (2009) confirmed that in the presence of lactose, transcription of the pathways for metabolizing maltose begins before maltose is detected. The dependency is asymmetrical, however. Exposure to maltose does not induce lactose utilization. Furthermore, in evolutionary experiments with a conditioning regime in which maltose does not follow lactose, over 500 generations *E. coli* loses the ability to predict the ecologically important sequence, but not the ability to utilize maltose. When maltose is detected, physiology is still modified for its metabolism in the evolved strains. What was lost during laboratory evolution, the researchers conclude, is “the asymmetrical cross talk between the two pathways” (p.221).

The third set of predictive experiments tested responses to QS in three species of proteobacteria with radically different lifestyles: *Burkholderia glumae,* a rice pathogen; *Burkholderia pseudomallei,* a human pathogen; and *Burkholderia thailandensis,* non-pathogenic saprophyte (Goo et al., 2012). QS signaling is an indicator of increasing cell density but could also predict the limits of population carrying capacity. In all three species, QS induced physiological preparation for stationary phase, survival of which requires the alteration of global metabolic processes. In contrast to QS-intact strains, QS mutants showed “massive and rapid population crashes,” which began shortly after commencement of the stationary phase. The cause of the population crash proved to be ammonia-related alkaline toxicity due to amino acid catabolism associated with stationary phase.

While both QS-competent and QS-mutant cells produced ammonia at roughly similar rates, the QS-competent strains were protected from catastrophe by QS-mediated production of oxalate, which counteracts base toxicity. Oxalate is produced at neutral pH, which suggests that QS-dependent “anticipatory production” of oxalate is not induced by a pH-dependent regulatory mechanism. The authors conclude the results “show that QS can function to allow anticipation of overcrowding and promote bacterial survival at maximum population carrying capacity, and provide insight into how QS bacteria have evolved to control both public and private goods,” namely, oxalate production and cell survival (Goo et al., 2012, p. 19778).

Based on these and other findings, Freddolino and Tavazoie (2012) assert that the homeostatic paradigm can no longer sustain an appropriate understanding of cellular behavior, whereas a ‘predictive-dynamic framework’ is more explanatory. They conclude (correctly, in my view) that regulatory networks in microbes and neural networks in metazoans have essentially the same function, and that microbiologists are now moving into behavioral territory previously occupied by animals with nervous systems. Whether these also share mechanisms with similar design principles remains to be seen.

A cautionary note must be sounded, however. Researchers in two of the studies described here (Tagkopoulos et al., 2008; Mitchell et al., 2009) claim their discoveries are demonstrations of associative learning. However, conditioning in these cases is clearly epigenetic. Whether this will count as ‘genuine conditioning’ remains an open question. Also, Tagkopoulos et al. (2008) claim that one of the implications of their research is that bacteria possess internal models or representations of the environment. The existence of representations and models, except in a metaphorical sense, is still open to debate even in humans (see, for example, Bechtel, 1998; van Gelder, 1998; Haselager et al., 2003), so microbiologists need to be careful.

### Putting it All Together: Signal Integration

So how does an individual bacterium integrate the information from a dizzying array of signaling pathways—sensorimotor, physiological, chemosensory, and communicative—into a coherent adaptative response? The short answer is we don’t know. This is perhaps the greatest challenge facing work in this field. We have already seen how cross talk can link substantially different signaling pathways (heat shock and respiration) to enable cells to predict regular changes in their habitat, but the mechanism is unknown. Mathematical or computational frameworks for modeling specific signaling pathways are proliferating, well, like bacteria in exponential growth (see, for example, Di Paola et al., 2004; Mehta et al., 2009; Liebal et al., 2010; Mitchell and Pilpel, 2011). How successful these proposals will prove remains to be seen.

At present prokaryotes offer very few examples of specialized information-processing ‘organs’ for investigation. The chemosensing receptor clusters at the leading pole of flagellated bacteria such as *E. coli* have already been suggested as analogous in important respects to neural clusters in metazoans with nervous systems (Stock et al., 2002). With an estimated 10,000 receptors in each cluster, with five different sensory targets and multiple binding sites, the computational complexity of the nanobrain is not especially tractable, however, (see **Box 2**). A second candidate involved in sensorimotor coordination are the FACs on the sides of *M. xanthus* together with the protein complex for transducing signals from the cell surface to the oscillator-regulator located in the cytosol. A third candidate organ for specialized information-processing in bacteria is the ‘stressosome,’ a large (multimillion dalton) protein complex associated with the general stress response that integrates multiple signals into a single outcome (σ^B^ activation), and is best-characterized in *B. subtilis*, although it is found many microbial phyla (Marles-Wright et al., 2008)^5^. Interestingly, the stressosome of *B. subtilis* recently has been found to be activated by a blue-light photoreceptor (van der Steen et al., 2013).

**Box 2. Implications for future research.**

**Object sensing.** James Shapiro described as “the greatest paper nobody has read.”^∗^ Dworkin’s (1983) report of the directed movement of *M. xanthus* swarms toward three-dimensional objects in multiple experiments. The swarms moved toward biologically salient objects (a clump of prey cells) but also toward chemically inert objects (sterilized glass beads). These experiments should be replicated, and, if the findings are confirmed, the mechanism investigated. It would be interesting to see whether previously uncharacterized photoreceptors are involved.

**Bistability.** Given the critical importance of bistability to cognition in more complex animals, what light can bistable regulatory networks in bacteria throw on the phenomenon, including its role in perception, learning and memory?

**Endogenous activity.** One of the greatest recent challenges to the classical computational view of cognition has been the discovery of endogenous, often oscillatory activity in the brain independent of external stimuli (Bechtel, 2012). Can oscillatory activity in regulatory circuits governing prokaryotic behavior tell us anything about the processes involved in endogenous brain activity?

**Nanobrain.** The cluster of chemoreceptors that governs motility (and other behaviors?) in *E. coli* provides a relatively tractable model for understanding how signals from multiple sources can be integrated into a behavioral response but still presents a monumental challenge.

**Valence.** Virtually nothing is known, in bacteria or humans, about how combinations of external stimuli of differing valence are integrated with interoceptive cues into a coherent behavioral response. Even more basically, hardly any information exists on the power spectrum (i.e. intensity and frequency) of signals to which any microorganism in any natural environment is exposed, except for circadian (24-h) frequency. Well-characterized stress responses in prokaryotes, particularly those in which multiple behavioral options exist, provide potentially productive platforms for investigation.

**Memory and learning.** Bacterial memory is yet to be properly investigated, and the mechanisms by which it occurs definitively characterized in different species. The possibility of associative learning in prokaryotes also remains terra incognita. *M. xanthus* may prove a good candidate for such exploration, given the unusually large number of genes (97) involved in producing serine/threonine protein kinases (STPK) in this diverse predator (Goldman et al., 2006). CaMKII is an STPK capable of autophosphorylation that is involved in multiple signaling cascades involved in homeostasis in animals, and also is thought to be important in human memory and learning. It would be interesting to see if there is any sequence homology between CaMKII and any of the myxobacterial STPKs.

**Communication.** The ability of QS molecules to participate in modifying the behavior of dispersed cells, particularly in relation to stress, has led to the suggestion that they may be precursors of hormonal messengers in animals, and indeed some homologies have been identified, for example, between AHLs and ghrelin (Tizzano and Sbarbati, 2006). Hormones, particularly those associated with the HPA axis, are major mediators of cognitive, affective and motive function in humans, (Vedder, 2008). The HPA axis, in turn, is the major mediator of the stress response in humans. Is there an evolutionary relation between QS molecules and the stress-induced molecular messengers of the mammalian brain, including not only hormones and neuropeptides but also immune elements such as cytokines?

*Shapiro made the remark in a workshop presentation attended by the author at the University of Exeter, in July 2006.

In actual fact, we have only begun to scratch the surface.

## Conclusion

While Jacques Monod’s famous assertion that “anything…true of *E. coli* must also be true of an elephant” (Friedmann, 2004) is an overstatement for many reasons, the Darwinian assumption that biological functions differ substantially ‘in form but not in kind’ continues to be born out in surprising ways, particularly since the advent of comparative genomics. Phylogenetic snobbery in the study of biological cognition is misguided, and for the same reasons such a bias would be in genetics or the study of respiration: there is much to be learned about the basics of biological functions and processes in ‘simple’ systems.

It is always satisfying, and the life sciences are seen to advance, when connections can be drawn between widely differing phyla with respect to important biological functions and processes. The study of cognition, for much of its existence, has proven resistant to this impulse. However, modern biology’s tools of genetic manipulation, comparative genomics, and molecular imaging techniques that enable the study of single behaving cells represent the means by which a continuum might be established, if it exists in fact.

Although knowledge of ST in both prokaryotes and eukaryotes has increased spectacularly in the interim due to technological and methodological innovations, interest in exploring the parallels between prokaryotic and neuronally based behavior has waned substantially since its heyday in the 1970s and 1980s. It is time to revive the project. The technical means available are better than they have ever been.

## Conflict of Interest Statement

The author declares that the research was conducted in the absence of any commercial or financial relationships that could be construed as a potential conflict of interest.

## Footnotes

1 According to Capra et al. (2006), approximately 20% of the human genome codes for proteins involved in signal transduction.

2 This paragraph is derived from personal communication with Jeffry Stock.

3 I am indebted to Klaas Hellingwerf for this observation in this form of words.

4 I am indebted to Klaas J. Jan Hellingwerf for this observation.

5 I am indebted to Klaas J. Jan Hellingwerf for this observation.
